# Supplementary material for: Germline variation contributes to false negatives in CRISPR-based experiments with varying burden across ancestries
Source: Nat Commun. 2024 Jun 7;15:4892. doi: 10.1038/s41467-024-48957-z (PMC11161638; doi:10.1038/s41467-024-48957-z)
Supplement: Supplementary file 1 — Supplementary Information [file 41467_2024_48957_MOESM1_ESM.pdf]

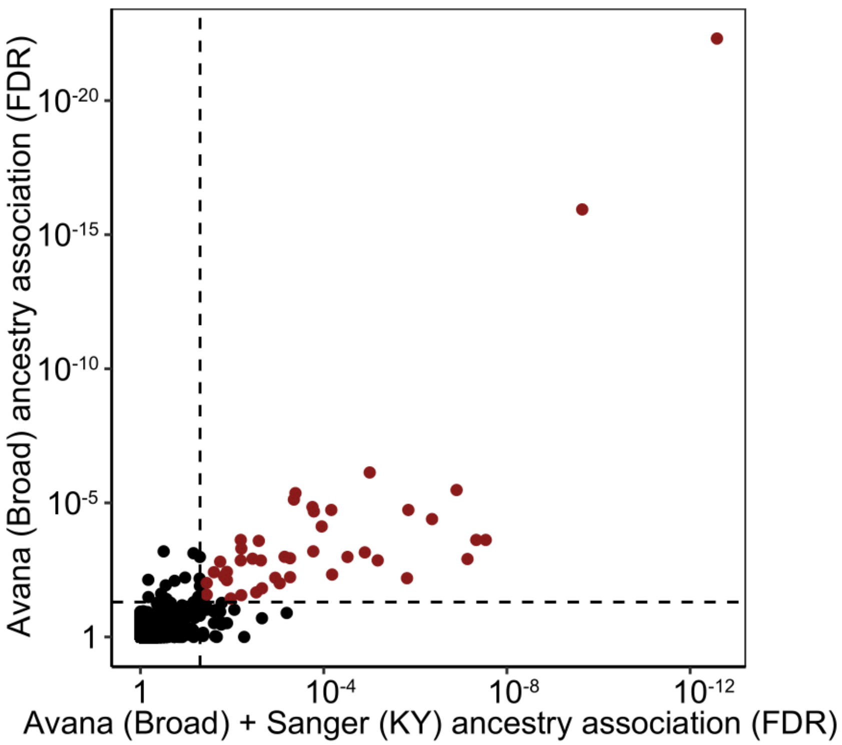

Supplemental Figure 1: Impact of pan-library harmonization on identification of ancestry associated dependencies. Ancestry-associated genetic dependencies were computed using the approach described in **Figure 1D** for both CRISPR libraries (x-axis, n = 611 samples), or for the subset of samples screened with the Avana library (y-axis, n = 558 samples). Raw data are described in Source Data S1

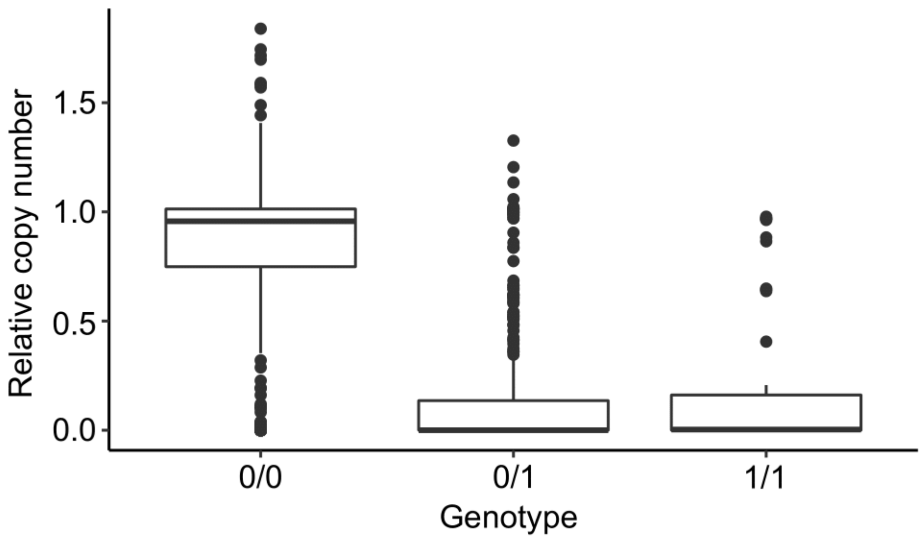

Supplemental Figure 2: Genotyping of the CDKN2B locus is confounded by copy number alterations at the CDKN2B locus. Cell lines were stratified by their genotype for the top CDKN2B d-QTL SNP and relative copy number at the CDKN2B locus was plotted for each sample bin. Raw data are described in source data S2. In the boxplot, the box includes the second and third data quartiles divided by a median line, and whiskers represent the first and fourth quartiles. Boxplot values for 0/0, 0/1, and 1/1 genotypes, respectively, are: median (9.57×10^{-1}, 3.09×10^{-5}, 2.97×10^{-3}); minimum (1.42×10^{-9}, 1.33×10^{-9}, 1.47×10^{-9}); maximum (1.83, 1.33, 0.98).

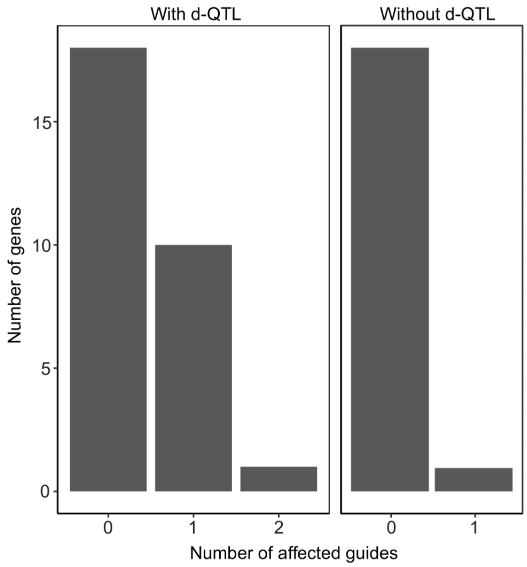

Supplemental Figure 3: Enrichment of mismatches in guides targeting genes with d-QTLs. Ancestry-associated genetic dependencies were stratified into those with a d-QTL (left) and those without (right). Genetic variants were mapped to the four guides targeting the dependency gene in question. Of the genes with genetic variants in at least one of the four guides, 11/12 are in guides targeting genes with a d-QTL.

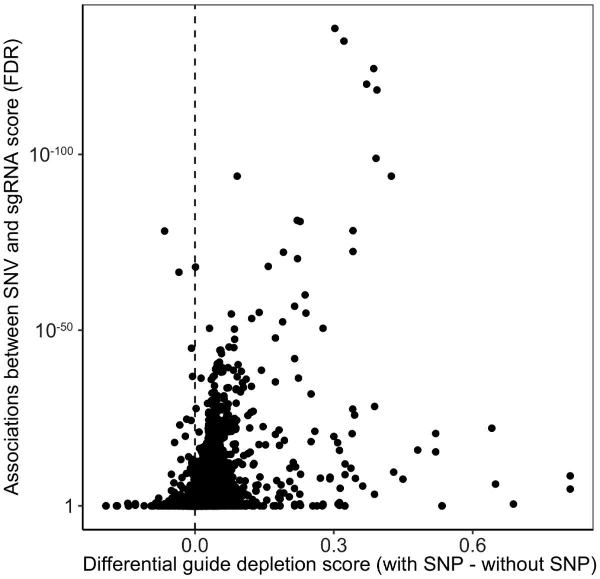

Supplemental Figure 4: Mismatches in sgRNA targeting sequences are protective against cutting activity. Mismatches in Avana sgRNA targeting sequences were identified for all Dependency Map cell lines profiled with both SNP6 genotyping and genome-scale CRISPR/Cas9 screens (n = 611). The association between mismatches and the sgRNA in question were computed with linear regression with correction for cancer cell lineage as a covariate. This analysis was restricted to include only guides in the Avana library. Nominal p-values were corrected for multiple hypothesis testing using the Benjamini-Hochberg procedure (y-axis). The difference in guide depletion scores between cell lines with and without a mismatch in the targeting sequence of the guide in question is indicated on the x-axis.

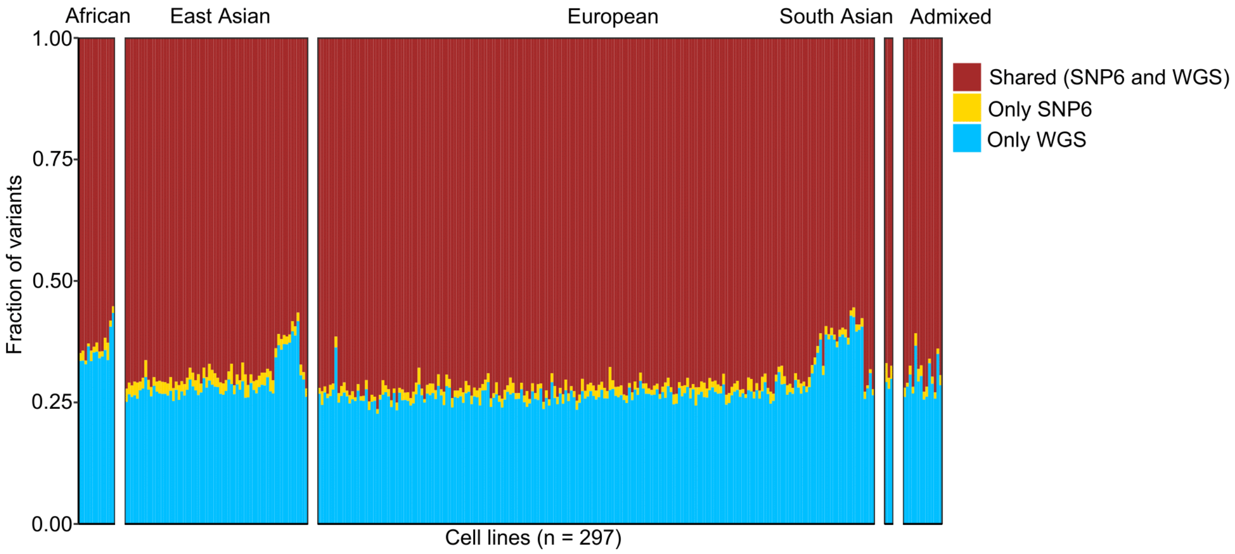

Supplemental Figure 5: Cell lines from individuals of recent African descent have an elevated rate of missed SNP6 genotypes. Cell lines (n = 297) were filtered to include those that have genotyping calls from both WGS and SNP6. Cell lines were binned by ancestry group from Figure 1B. The fraction of variants in each cell line that were called by only WGS (blue), only SNP6 (yellow), or both SNP6 and WGS (red) are indicated. Raw data are described in Source Data S5.

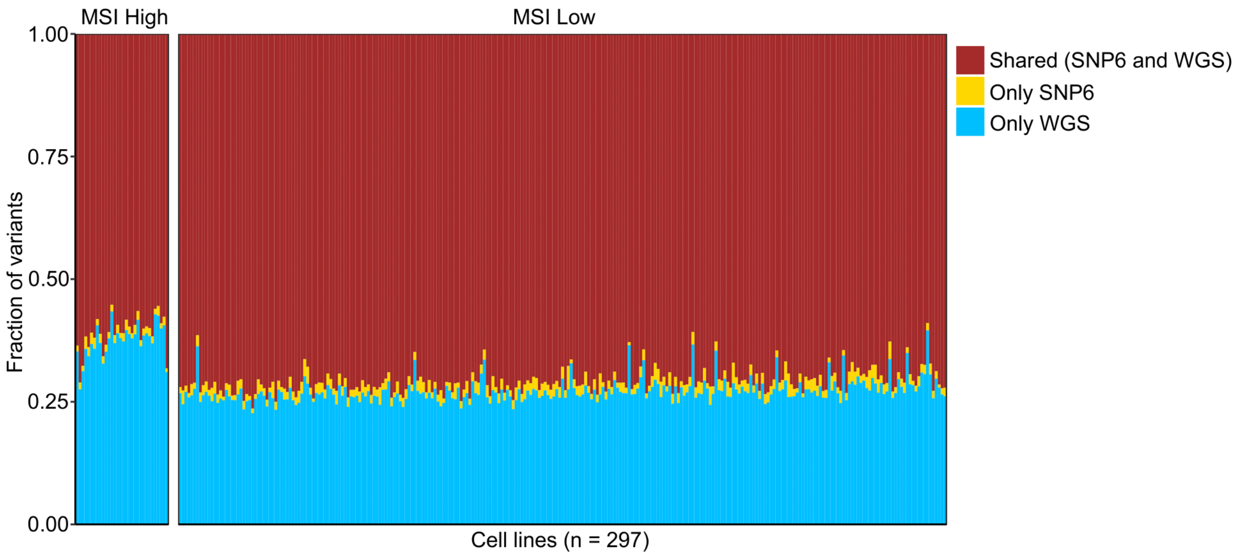

Supplemental Figure 6: Samples with microsatellite instability have an increased number of variants called exclusively by WGS. Variant mapping was performed as described in Supplemental Figure 4 for 297 cell lines, except samples were stratified by MSI status. Raw data are described in Source Data S5.

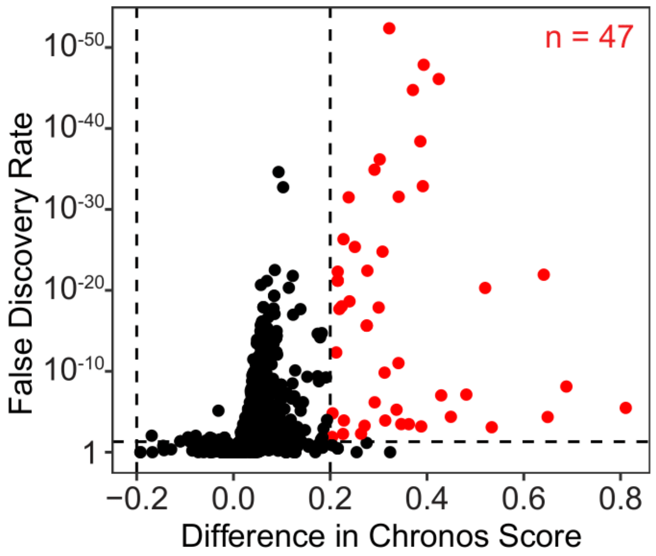

Supplemental Figure 7: Association between gene-level Chronos scores and SNV mismatches in Avana guide targeting sequences. For all genes profiled in the Dependency Map, cell lines (n = 611) were stratified into cell lines with a mismatch in one or more guides targeting the gene of interest, and cell lines with mismatches in zero of the four guides. The association between mismatch status and the aggregate gene-level (Chronos) dependency score for each gene was computed using linear regression with correction for cancer cell lineage as a covariate. Nominal p-values were corrected for multiple hypothesis testing using the Benjamini-Hochberg procedure (y-axis). The differential Chronos score between cell lines with and without a variant present in the targeting sequence of one guide is indicated on the x-axis. Statistically significant associations (red points, n = 47) are those with a false discovery rate < 0.05 and a Chronos score differential greater than 0.2. Raw data are described in Source Data S6.

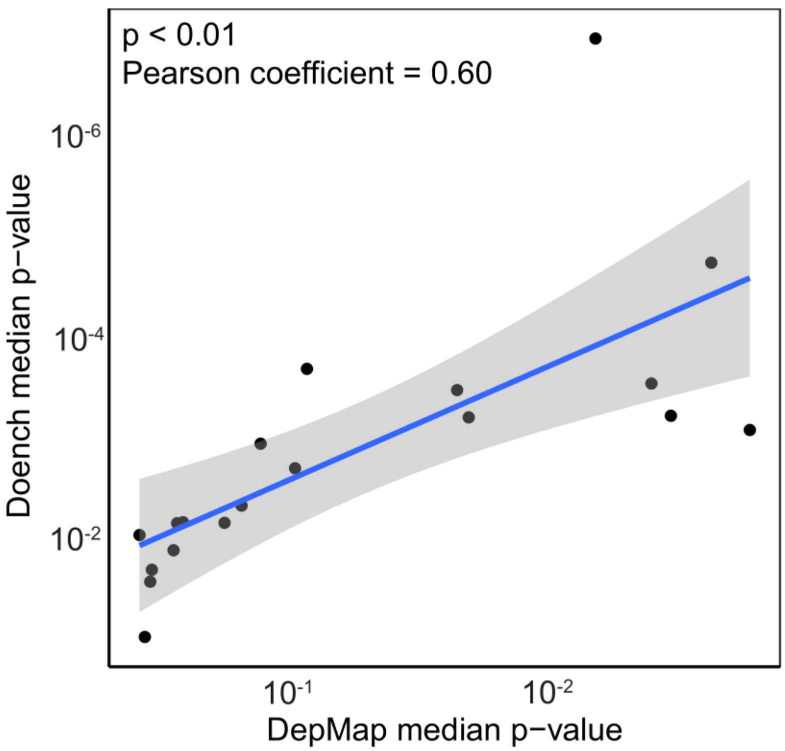

Supplemental Figure 8: Correlation between mismatch position and strength of association with sgRNA depletion score. Linear regression between the positional associations computed in this study and those computed in Doench et al[1] Figure 3E. Linear regression was performed for n = 20 guide positions, Pearson correlation coefficient = 0.60, $p < 0.01$.

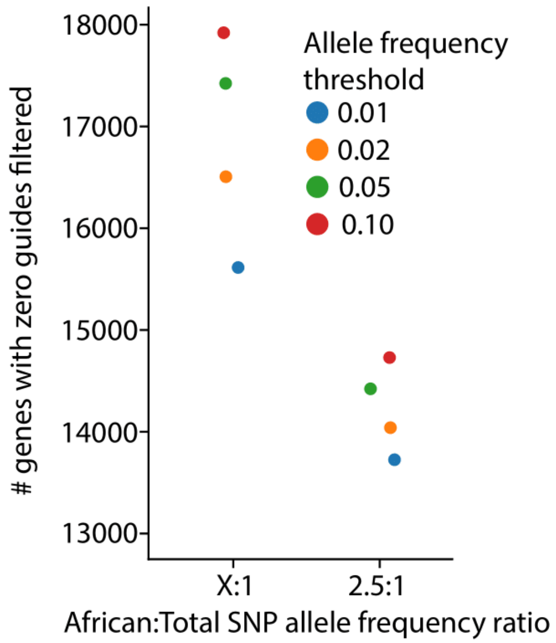

Supplemental Figure 9: Impact of mismatch filtering on sgRNA design. CRISPR/Cas9 guides with canonical NGG PAM sites were designed using the CRISPick algorithm. Guides were filtered to exclude those with gnomAD variants at the indicated rate in the total population (colored dots) or those that are 2.5x higher in African individuals (x-axis).

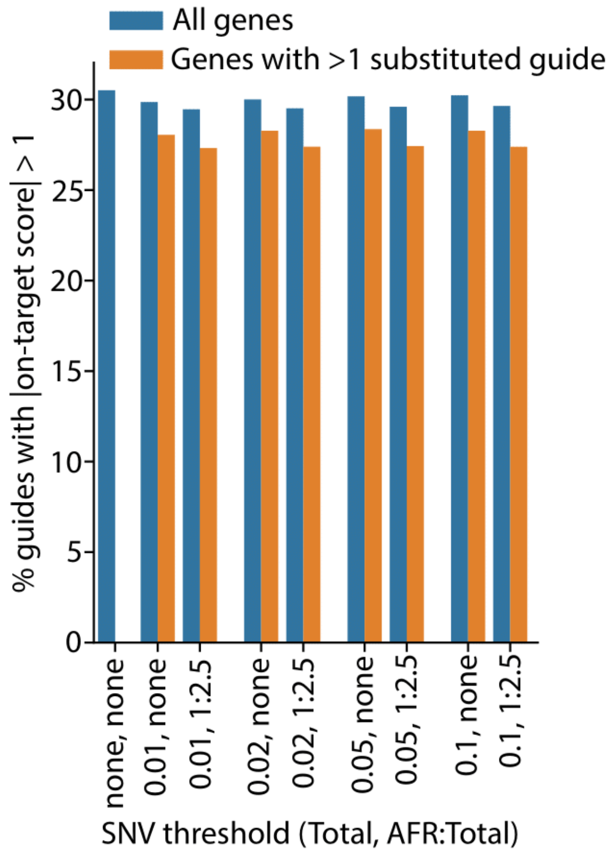

Supplemental Figure 10: Impact of mismatch filtering on CRISPR/Cas9 guide quality scores. Guides were designed and filtered as described in **Supplemental figure 8.** On-target scores, a metric for the likelihood of on-targeting genome editing, was computed for either all genes (blue) or genes where one or more guides exceeded the indicated mismatch filtering threshold (orange).

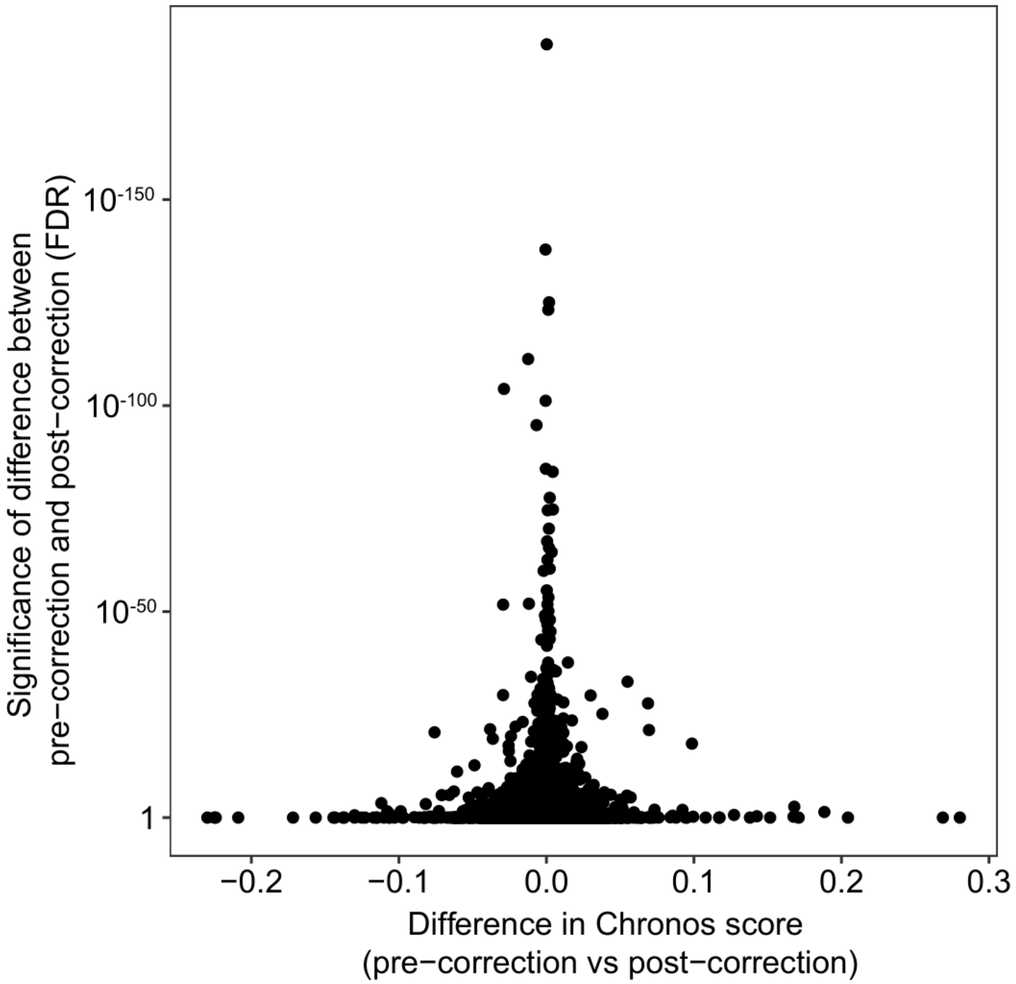

Supplemental Figure 11: Impact of variant correction on Dependency Map gene-level (Chronos) dependency scores. Correction for variation in sgRNA targeting sequences was implemented in the Dependency Map as described in the Methods section. Median gene dependency (Chronos) scores were computed across all cell lines and the difference in scores (pre-correction vs post-correction) is plotted on the x-axis. Nominal p-values between the pre- and post-correction datasets for each gene were computed using a Wilcoxon test and were adjusted for multiple hypothesis testing using the Benjamini-Hochberg procedure. Raw data are described in Source Data S11

Supplementary References:

1. Doench, J. G. *et al.* Optimized sgRNA design to maximize activity and minimize off-target effects of CRISPR-Cas9. *Nat. Biotechnol.* **34**, 184–191 (2016).
