# Supplementary material for: Germline variation contributes to false negatives in CRISPR-based experiments with varying burden across ancestries
Source: Nat Commun. 2024 Jun 7;15:4892. doi: 10.1038/s41467-024-48957-z (PMC11161638; doi:10.1038/s41467-024-48957-z)
Supplement: Supplementary file 3 — Reporting Summary [file 41467_2024_48957_MOESM3_ESM.pdf]

nature portfolio

Corresponding author(s): Jesse Boehm, Rameen Beroukhim

Last updated by author(s): Feb 14, 2024

Reporting Summary

Nature Portfolio wishes to improve the reproducibility of the work that we publish. This form provides structure for consistency and transparency in reporting. For further information on Nature Portfolio policies, see our Editorial Policies and the Editorial Policy Checklist.

Statistics

For all statistical analyses, confirm that the following items are present in the figure legend, table legend, main text, or Methods section.

n/a	Confirmed	
☐	☒	The exact sample size (*n*) for each experimental group/condition, given as a discrete number and unit of measurement
☒	☐	A statement on whether measurements were taken from distinct samples or whether the same sample was measured repeatedly
☐	☒	The statistical test(s) used AND whether they are one- or two-sided<br>*Only common tests should be described solely by name; describe more complex techniques in the Methods section.*
☐	☒	A description of all covariates tested
☐	☒	A description of any assumptions or corrections, such as tests of normality and adjustment for multiple comparisons
☐	☒	A full description of the statistical parameters including central tendency (e.g. means) or other basic estimates (e.g. regression coefficient) AND variation (e.g. standard deviation) or associated estimates of uncertainty (e.g. confidence intervals)
☐	☒	For null hypothesis testing, the test statistic (e.g. *F*, *t*, *r*) with confidence intervals, effect sizes, degrees of freedom and *P* value noted<br>*Give P values as exact values whenever suitable.*
☒	☐	For Bayesian analysis, information on the choice of priors and Markov chain Monte Carlo settings
☒	☐	For hierarchical and complex designs, identification of the appropriate level for tests and full reporting of outcomes
☐	☒	Estimates of effect sizes (e.g. Cohen's *d*, Pearson's *r*), indicating how they were calculated

Our web collection on statistics for biologists contains articles on many of the points above.

Software and code

Policy information about availability of computer code

Data collection	No software was used for data collection in this manuscript. All data used in these studies is publicly available. Custom code was used for all analyses described in this manuscript. All code is deposited in the following github repository: https://github.com/beroukhim-lab/ancestry_manuscript_code.
Data analysis	No commercial software was used to analyze the data in this study. The following open source software was used to analyze the data in this study: bcftools (v1.16), Eagle (v2.4), Minimac4 (v1.6.6), RFMix (v2.0), R (v4.2.x). A custom python script (https://software.broadinstitute.org/cancer/cga/contest_prepare2) was used to convert birdseed files into the vcf file format. All other custom code was written in R(v4.2.x). Custom code written for this manuscript is deposited in the following github repository: https://github.com/beroukhim-lab/ancestry_manuscript_code.

For manuscripts utilizing custom algorithms or software that are central to the research but not yet described in published literature, software must be made available to editors and reviewers. We strongly encourage code deposition in a community repository (e.g. GitHub). See the Nature Portfolio guidelines for submitting code & software for further information.

Data

Policy information about availability of data

All manuscripts must include a data availability statement. This statement should provide the following information, where applicable:

- Accession codes, unique identifiers, or web links for publicly available datasets
- A description of any restrictions on data availability
- For clinical datasets or third party data, please ensure that the statement adheres to our policy

The input and intermediate data for figure generation are deposited on github (https://github.com/beroukhim-lab/ancestry_manuscript_code) or on figshare (https://figshare.com/projects/Germline_variation_contributes_to_false_negatives_in_CRISPR-based_experiments_with_varying_burden_across_ancestries/202215). The DepMap data was accessed from the DepMap web portal (depmap.org) and download links are provided in the github README file (https://github.com/beroukhim-lab/ancestry_manuscript_code). The gnomAD (v3.1.2) datasets were downloaded from the gnomAD website (gnomad.broadinstitute.org). The CCLE SNP6 genotyping files were downloaded from14. DepMap WES/WGS data were downloaded from the DepMap web portal (depmap.org). The list of COSIC (v98) genes was downloaded from the COSMIC data portal (cancer.sanger.ac.uk.cosmic). TCGA somatic mutation MAF files were accessed from the GDC Data Portal (https://portal.gdc.cancer.gov) and germline mutations were accessed from a previously published study45. The publicly available CRISPR guide efficacy data from Doench et. al.3 are included as supplemental data in the associated manuscript (https://www.nature.com/articles/nbt.3437#Sec24). The publicly available CRISPR guide map data from Sanson et. al.27 are included as supplemental data in the associated manuscript (https://www.nature.com/articles/s41467-018-07901-8#Sec26). The publicly available data gnomAD genotyping data from Koenig et. al.44 are available on the gnomAD website (gnomad.broadinstitute.org). The publicly available TCGA germline variant data used in this study are available on the ISB cancer genome cloud and can be accessed with the following procedure (https://gdc.cancer.gov/about-data/publications/PanCanAtlas-Germline-AWG)45. Guide map matrices for Avana, Calabrese, Custom, Dolcetto, Gecko, Sanger, and TKO libraries are available on figshare (https://figshare.com/projects/Germline_variation_contributes_to_false_negatives_in_CRISPR-based_experiments_with_varying_burden_across_ancestries/202215). Cell line metadata for DepMap cell lines was downloaded from the DepMap data portal (see https://github.com/beroukhim-lab/ancestry_manuscript_code). The remaining source data are available within the Article, Supplementary Information, or Source Data file.

Research involving human participants, their data, or biological material

Policy information about studies with human participants or human data. See also policy information about sex, gender (identity/presentation), and sexual orientation and race, ethnicity and racism.

Reporting on sex and gender	Findings in this paper apply to all sexes and genders. Sex and gender were not included as part of the analysis described in this paper and were not considered in study design. This study used preexisting publicly available data; sex information was collected as part of the gnomAD dataset and is reported in the associated sample metadata, ancestry inference was performed on all samples in the Cancer Dependency Map and is reported in the associated sample metadata.
Reporting on race, ethnicity, or other socially relevant groupings	This manuscript uses the term 'ancestry' to broadly describe the continental ancestry of individuals whose genomes were sequenced as part of this study. We recognize, and discuss in the manuscript, the limitations of broadly categorizing individuals based on genetic ancestry. This manuscript is careful to not use the terms race or ethnicity to describe genetic ancestry.
Population characteristics	This study used genotyping data from all unrelated individuals profiled in gnomAD.
Recruitment	No participants were recruited for this study.
Ethics oversight	No approval was required for this study.

Note that full information on the approval of the study protocol must also be provided in the manuscript.

Field-specific reporting

Please select the one below that is the best fit for your research. If you are not sure, read the appropriate sections before making your selection.

☒ Life sciences ☐ Behavioural & social sciences ☐ Ecological, evolutionary & environmental sciences

For a reference copy of the document with all sections, see nature.com/documents/nr-reporting-summary-flat.pdf

Life sciences study design

All studies must disclose on these points even when the disclosure is negative.

Sample size	We performed a power calculation to determine the likelihood of detecting statistical signal in a subset of analyses described in this manuscript. We did not perform any experiments or analyses in this manuscript that necessitated performing a power calculation to determine sample size.
Data exclusions	This paper used CRISPR screening data from the Cancer Dependency Map. Samples which were not profiled with SNP6 genotyping arrays (PMID: 31068700) were excluded from analysis.
Replication	Replicate experiments were not attempted in this study. All analysis in this paper are on preexisting publicly available datasets. This study uses

Replication	CRISPR screening data generated as part of the DepMap project, which included technical replicates. However this study did not generate any new genome-scale CRISPR screening data.
Randomization	Sample randomization was not performed in this study. Randomization is not relevant to this study because most analyses stratify samples into classes (e.g. ancestry or genotype status). When appropriate, covariates (like cancer lineage) are corrected for by adding additional terms to the linear regression. These covariates are amongst the data listed in supplemental table 11.
Blinding	Investigators were not blinded to group allocation during data collection and analysis. Blinding was not relevant to this study because this study used preexisting publicly available data and all analyses were performed in an unbiased manner. No data was collected as part of this study, and thus there was no situation in which blinding was appropriate.

Reporting for specific materials, systems and methods

We require information from authors about some types of materials, experimental systems and methods used in many studies. Here, indicate whether each material, system or method listed is relevant to your study. If you are not sure if a list item applies to your research, read the appropriate section before selecting a response.

Materials & experimental systems

n/a	Involved in the study
☒	☐ Antibodies
☒	☐ Eukaryotic cell lines
☒	☐ Palaeontology and archaeology
☒	☐ Animals and other organisms
☒	☐ Clinical data
☒	☐ Dual use research of concern
☒	☐ Plants

Methods

n/a	Involved in the study
☒	☐ ChIP-seq
☒	☐ Flow cytometry
☒	☐ MRI-based neuroimaging

Plants

Seed stocks	*Report on the source of all seed stocks or other plant material used. If applicable, state the seed stock centre and catalogue number. If plant specimens were collected from the field, describe the collection location, date and sampling procedures.*
Novel plant genotypes	*Describe the methods by which all novel plant genotypes were produced. This includes those generated by transgenic approaches, gene editing, chemical/radiation-based mutagenesis and hybridization. For transgenic lines, describe the transformation method, the number of independent lines analyzed and the generation upon which experiments were performed. For gene-edited lines, describe the editor used, the endogenous sequence targeted for editing, the targeting guide RNA sequence (if applicable) and how the editor was applied.*
Authentication	*Describe any authentication procedures for each seed stock used or novel genotype generated. Describe any experiments used to assess the effect of a mutation and, where applicable, how potential secondary effects (e.g. second site T-DNA insertions, mosiacism, off-target gene editing) were examined.*
